# The ParentingWell Learning Collaborative Feasibility Study: Training Adult Mental Health Service Practitioners in a Family-Focused Practice Approach

**DOI:** 10.1007/s10597-021-00818-5

**Published:** 2021-04-04

**Authors:** Joanne Nicholson, Kelly English, Miriam Heyman

**Affiliations:** 1 Institute for Behavioral Health, The Heller School, Brandeis University, 415 South Street, Waltham, MA 02453 USA; 2 Children’s Behavioral Health Knowledge Center, Massachusetts Department of Mental Health, 25 Staniford Street, Boston, MA 02114 USA; 3 The Lurie Institute for Disability Policy, The Heller School, Brandeis University, 415 South Street, Waltham, MA 02453 USA

**Keywords:** Parents with mental illness, Family-focused practice, Learning collaborative, Practitioner training

## Abstract

This study investigates the feasibility and impact of the ParentingWell Learning Collaborative (PWLC) model in supporting mental health practitioners in implementing the family-focused ParentingWell practice approach with adults with mental illness. An exploratory design and qualitative methods were employed. Practitioners (*n* = 29) participated in in-person orientation, training and debriefing sessions; virtual coaching sessions; and via an interactive online hub. Researchers obtained data on participant engagement and satisfaction, and participants’ reports of use, helpfulness, intention to use and impact. Participants were engaged in and highly satisfied with the PWLC. They deployed PWLC skills, tools and resources with parents. Evidence of impact was provided at the personal, practice and organizational levels. This study provides preliminary support for the feasibility and impact of the PWLC. Clear specification of a theoretically-based training model for practitioners is an essential step in adapting, implementing and testing interventions in new contexts .

## Introduction

Individuals with mental illness in the US are as likely as other adults to be parents (Nicholson et al., 2004). Parenthood is a major life activity and a significant role that promotes and benefits from engagement and participation in community life. Parents with mental illness are extremely vulnerable to the loss of contact with or custody of their children (Friesen et al., 2009; Kaplan et al., 2019; Powell et al., 2020; Powell & Nicholson, 2019). “Failure” in the parenting role, whether attributed by others when custody is lost or perceived by parents themselves, when they feel “ineffective” or disengaged from children, undermines community engagement for adults and, likely, negatively impacts health and function, and employment (Reupert et al., 2015b). Parental mental illness can have multifaceted consequences for children, influencing both their physical and mental health (Brockington et al., 2011; Campo et al., 2020; Dean et al., 2010, 2018). The impact of parental mental illness for both adults and children is undoubtedly magnified when illness goes untreated and parents do not receive appropriate, accessible support (Reupert & Maybery, 2007).

The experiences of parents have been explored, their concerns and needs documented, and rehabilitation resources and interventions developed and pilot-tested (Biebel et al., 2014; Nicholson, 2009; Nicholson & Blanch, 1994; Nicholson & Henry, 2003; Nicholson et al., 2009, 2014, 2016). The needs of parents, however, remain frequently unidentified and unmet in clinical practice, contributing to poor outcomes for adults and their families. Few interventions have specifically targeted the impact of parenting and family life on adults with serious mental illness (Nicholson et al., 2009, 2016; Reupert et al., 2017; van der Ende et al., 2014). Other interventions (e.g., supported employment, supported housing) may be person-centered, but do not explicitly consider an individual’s needs as a parent (Nicholson, 2014). Consequently, opportunities for social connection, family support, and community participation are overlooked or outright impeded, not only when parents and their children are young, but across the lifespan. While the desire to raise children well may provide motivation for change (e.g., seeking treatment, improving life circumstances), the stresses of parenting, when poorly managed, may have negative impact on recovery (Reupert et al., 2015b).

The mental health services workforce (e.g., psychologists, social workers, psychiatric nurses, counselors, case workers, peer specialists) is ill-prepared to work effectively with adults who are parents (Adderley et al., 2020). There are many obstacles to family-focused, parent-informed practice, professional development, and practice improvements. Issues identified in the research literature include failure to recognize the parenting role and its significance for adults, practitioners’ attitudes and expectations, limited skills, the lack of best practices and practice guidelines, and challenges in interagency collaboration (David et al., 2011; Lauritzen et al., 2015; Maybery & Reupert, 2006, 2009; Nicholson et al., 2015; Skogøy et al., 2019). The uptake and sustainability of family-focused practice with adult clients who are parents is further influenced by organizational, practitioner and parent-client factors (Allchin et al., 2020). Change at the systems level is likely required to ensure integrated supports for families (Isobel et al., 2019). Adderley and colleagues suggest that future efforts focus on practitioners’ skills, knowledge and confidence in working in family-focused ways (Adderley et al., 2020). In doing so, organizational and community context must be considered, at the least.

Efforts have been made to bring attention to and address workforce challenges through professional development. The “Keeping Families and Children in Mind” initiative was developed through a review of existing workforce packages and a Delphi process to identify themes for the core modules of the resource, resulting in interactive, web-based material to be accessed by individuals or in a facilitator-led training group (Reupert et al., 2011). In a mixed methods study of the training, participants expressed positive views of the resource and increased knowledge and awareness of the importance of talking with all family members, and saw the potential for implementing the resource within their organization. A subsequent study of the Australian e-learning resource for the Let’s Talk about Children (LTC) intervention provides evidence of change in professionals’ self-reports of family and parenting support, assessing impact on the child, connectedness, and parenting and mental illness; participants described becoming more family responsive as a result of the training (Tchernegovski et al., 2015). In a related review of e-learning professional development resources for families where a parent has a mental illness, authors summarize common features, issues and techniques across a number of available e-learning packages, acknowledging the critical importance of focusing on the learning process (Reupert et al., 2015a). They suggest that the evaluation of the effectiveness of e-learning resources is undermined by inadequate study design or measurement. Evaluation of professional development resources is likely further undermined by the lack of a clear model of program theory and, consequently, the lack of well-specified model components, both of which would contribute to selection of appropriate measures and outcomes. Training resources require specification in much the same way as the interventions for which they are developed, when training is framed as intervention at the practice level.

An additional consideration in preparing the workforce is that a portion of the workforce has experience with mental illness, whether disclosed or not. The emergence of the peer specialist role and the growing emphasis on employing individuals with lived experience convey significant advantages as well as potential challenges (Farkas & Boevink, 2018; Gagne et al., 2018; Repper & Carter, 2011; Rogers & Swarbrick, 2016). Sensitive topics may trigger responses in practitioners with lived experience as well as in parents. However, training, coaching and supervisory resources may not be developed or implemented with the needs of staff members or parents with mental illness in mind (David et al., 2011). To our knowledge there are no training and support materials designed specifically to promote peer support with parents; parenting and family topics, for example, may not be routinely covered in the training of peer specialists (Nicholson & Valentine, 2018, 2019). This underscores the importance of developing resources and tools for training, coaching, feedback and ongoing support that are relevant and accessible to practitioners, including those with lived experience, their colleagues and supervisors.

### The ParentingWell Practice Profile

The focus of our larger program of research is to address the gap in parent- and family-informed practice in adult mental health services in the US. The ParentingWell Practice Profile (Nicholson & English, 2019) was developed in the context of a state-wide initiative to adapt the evidence-based Let’s Talk model, originally developed and tested in Finland (Solantaus et al., 2009, 2010, 2015), and replicated for testing in Australia (Maybery et al., 2019) and Greece (Giannakopoulos et al., 2021), to the US and Massachusetts service delivery context. Adding a parenting intervention or approach to standard treatment has been found to improve the parenting capacity of people with personality disorder, for example, while supporting practitioner’s capacity to work with this population (Gray et al., 2019).

A practice profile describes the program or practice approach, including essential functions, operational definitions, and practical performance strategies (Practice Profile Planning Tool, n.d.). The ParentingWell practice approach is based on and aligns with Self-Determination Theory (Deci & Ryan, 2012), with core elements of Engage and Explore (*autonomy*: identifying personal circumstances and motivation), Plan (*competence*: setting goals, assessing progress, and building self-efficacy), and Access and Advocate (*relatedness*: linking to social and professional supports and resources). Practice principles underlying the approach include the principles of family-focused, culturally-sensitive, strengths-based and trauma-informed practice. We anticipate proximal parent outcomes of ParentingWell practice, based on prior pilot studies, to include improved well-being and functioning; reduced stress; enhanced self-efficacy, hope and optimism; and an improved therapeutic alliance (Nicholson et al., 2009, 2014, 2016). Distal outcomes include an enhanced parent–child relationship and, ultimately, improved outcomes for children.

The steps in adapting an intervention for implementation and testing in a new context have been elaborated, highlighting the importance of stakeholder involvement, the specification of core elements and mechanisms, and the assessment of available resources and infrastructure (Card et al., 2011; Escoffery et al., 2018, 2019; Movsisyan et al., 2019). While training staff is an essential step in the process, staff training is the least well-articulated and less frequently mentioned step; training modifications are much less commonly reported (Card et al., 2011; Escoffery et al., 2018, 2019). What is missing in prior research and implementation work is a theoretically-sound learning opportunity for practitioners, who could be encouraged to adopt and use stakeholder-informed, empirically-based resources in the context of their home agencies (Adderley et al., 2020). Our larger mission is to lay a foundation of well-articulated program theory, not only for the ParentingWell practice approach itself, but to provide an explicit, empirically-sound strategy for adapting, implementing and sustaining an evidence-based approach to practitioner training to support the implementation and rigorous testing of the ParentingWell practice approach (Davies et al., 2010; Hoffman et al., 2014; Movsisyan et al., 2019).

### The ParentingWell Learning Collaborative

Learning collaborative models are often targeted to practitioners to promote the implementation of evidence-based practices (Nadeem et al., 2014, 2016). Researchers have highlighted the need for the development and testing of theoretically-based, manualized learning collaborative models, particularly that meet the needs of public mental health organizations (Nadeem et al., 2016). The core process of learning collaboratives typically includes access to experts in the field and activities informed by adult learning principles to engage participants and promote reflection and the transfer of learning into actual practice (Nadeem et al., 2016). In-person learning sessions, Plan-Do-Study-Act cycles, multidisciplinary teams, coaching and interest group calls are common components. Interactive, experiential training and observation, coaching and ongoing support approaches have been found to be most effective in changing practice behavior and implementing evidence-based practices (Ghate, 2016).

The Theory of Planned Behavior (TPB) has provided the frame for numerous studies of individual health behavior change as well as practice change per se among mental health practitioners (Asare, 2015; Britt et al., 2011; Brouwer, 2009; Casper, 2007; DeMaria et al., 2019; Jokonya, 2017). TPB suggests that the intention to change behavior, considered the best predictor of a deliberate behavior, is a function of attitudes toward the behavior, subjective norms, and perceived control over the behavior (Ajzen, 1991; Casper, 2007). Attitudes reflect beliefs about the outcomes associated with performing a behavior. Subjective norms refer to how others would judge a person for performing the behavior. Perceived control is the self-assessment of the capability or skill and opportunity to perform the behavior (Casper, 2007). Changes in attitudes, norms and perceptions of behavioral control should contribute to changes in intentions and, under the right circumstances, should result in actual change or use of a new skill or practice (Jokonya, 2017). The Theory of Planned Behavior does not, by itself, result in practice change and intervention uptake. However, it does provide the first step (i.e., specifying the core elements or training targets) for linking theory with empirically-based behavior change strategies (i.e., core activities/theory of action) (Bajpai et al., 2019). Effective behavior change strategies have been inventoried and summarized across domains, as researchers and clinicians work to facilitate intervention development, research and communication (Abraham & Michie, 2008).

**Fig. 1:**
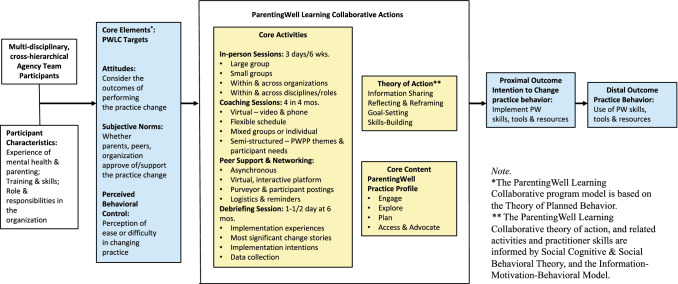
ParentingWell learning collaborative for practice change

In Fig. 1, ParentingWell Learning Collaborative (PWLC) core elements, and intervention targets informed by the Theory of Planned Behavior (TPB) (Ajzen, 1991; Casper, 2007; Jokonya, 2017), are linked with effective behavior change techniques (Abraham & Michie, 2008), and translated into the PWLC theory of action with related activities (see Fig. 1).

### The Study Goal

The specific goal of this study is to investigate the feasibility and explore the preliminary impact of the ParentingWell Learning Collaborative model in supporting practitioners of all types (e.g., social work clinicians, mental health counselors, peer specialists) in scaling out and scaling up a new practice approach to working with adults with mental illness who are parents (Movsisyan et al., 2019). Feasibility studies are conducted to inform the development or refinement of implementation strategies, and to facilitate the identification of change mechanisms (Abbott, 2014; Arain et al., 2010; Pearson et al., 2020). Key foci may include the willingness of clinicians to participate in implementing and testing an intervention, whether an intervention can be delivered as intended, and the identification of clinician training needs and competence (Abbott, 2014). Modifications or adjustments can then be made to the design and methods of subsequent, larger-scale, rigorous intervention testing. In the context of this study, feasibility reflects whether implementation and use of the proposed model for training and supporting practitioners is practical to use in the context of the practice environment and, ultimately, whether it will achieve the desired impact, that is, whether it benefits its intended users and the parents they serve.

Research questions for the present study include: (1) Is a learning collaborative model a feasible way to train and coach diverse practitioners in a new practice approach to working with parents with mental illness? and (2) What is the preliminary impact of the ParentingWell Learning Collaborative on practitioners and parents? Feasibility is assessed in this study in terms of practitioner *engagement*, and practitioner self-reports of *satisfaction* and ratings of *use*, *helpfulness* and *intention to use* ParentingWell skills, tools and resources in the future. Preliminary impact is explored as it relates to content reflecting the core elements of the ParentingWell approach (i.e., Engage, Explore, Plan, and Access and Advocate) and practitioner-reported shifts in constructs informed by the Theory of Planned Behavior (i.e., *attitudes, norms, perceived behavioral control* and *intention to change practice*) and actual examples of practice change with parents, provided by practitioners themselves. Findings will inform further refinements to the PWLC model, fidelity considerations, and the development of strategies for measuring practice attitudes, competence and confidence in future research, as well as investigating the longer-term impact of the ParentingWell practice approach (e.g., outcomes for parents and families).

## Methods

An exploratory design and mixed methods approach were employed to assess the feasibility and preliminary impact of the ParentingWell Learning Collaborative (PWLC). Qualitative methods are well-suited to an exploratory study, for which standardized measures may not be available or appropriate, to develop an initial understanding in an unexplored area (Creswell & Poth, 2016; Levitt et al., 2018).

### The Sample and Recruitment Process

Participants were recruited from provider agencies across Massachusetts serving adults with mental illness in March 2019. An invitation email was sent to agencies listed as mental health service vendors by the Massachusetts Department of Mental Health. Agencies were invited to submit a team of three to five staff members, at least one of whom had to be a supervisor or program manager, responsible for leading and facilitating the implementation in the agency context. Staff members included clinicians, peer specialists, or case managers. All team members must have been co-located at a single site/location and work within a single program area or level of care. Participating practitioners must have been able to identify parents or adults who were planning to become parents with whom they were working. All agencies had to identify a senior leader who served as executive sponsor for the initiative (e.g., a CEO, Executive Director, COO, Division Director, VP). This person was expected to attend the onsite orientation, watch the video overview webinar, and serve as champion within the agency for ongoing support of the initiative. Applications were reviewed by the project team. A sample of 30 participants was recruited representing five agencies, one of which was a regional Department of Mental Health (DMH), the state mental health authority.

### The ParentingWell Learning Collaborative Procedures

#### The Orientation Process

Following the application process, an orientation session was held at each participating agency site in April and May 2019 with agency leaders and 3 to 5 staff members who would be participating in the PWLC. An overview of the PWLC was provided, along with a clear explanation of expectations for participant teams. Participants were given copies of the ParentingWell Practice Profile and the link to the introductory webinar, which they were encouraged to view prior to the first training session; and access to the PW Basecamp online project hub, where training schedules, materials and resources were posted, as well as prompts for discussion and resource sharing. At the orientation session, participants completed background surveys, and jotted down their learning intentions. The first two authors, doctoral level, licensed, experienced clinical professionals, facilitated all PWLC sessions, with assistance from participants as relevant (e.g., co-leading role play sessions). All research procedures and measures were administered by another research staff member, working independently from the trainers to provide oversight for all data collection and management. An additional member of the research staff participated in the analysis and interpretation of data and served as a co-author in preparing this paper.

#### In-Person Training Sessions

Three in-person, full-day PWLC training sessions were held in May and June 2019. PWLC trainings and coaching content reflected the core elements of the ParentingWell Practice Profile: Engage, Explore, Plan, and Access and Advocate. Participants were encouraged to reflect, rehearse, take the perspective of the parent, conduct an agency scan or systematic review of agency policies and procedures, consider their routine practice, share their lived experience, complete activities and worksheets, set SMART goals, consider their own support network, respond to typical family scenarios, map resources, and debrief on lessons applied in their agency contexts. Video clips provided explanations of underlying theory and examples of parents sharing their stories, and online games (e.g., “Kahoots”) were used to encourage participants to question assumptions. A training session was devoted to considering implementation issues, discussing workplace challenges, and committing to plans to “keep it going” (i.e., sustainability) in the agency environment.

#### Virtual Project Hub

A ParentingWell virtual project hub was created using Basecamp, a web-based project management and team communication tool. Project training agendas and materials were available, as well as an ongoing message board with posts from trainers and participants regarding the scheduling of project events and the sharing of resources. Participants could register for coaching sessions and check on logistics for in-person meetings. The “Campfire” space provided an opportunity for trainers and participants to interact asynchronously via posts and responses.

#### Coaching Sessions

Coaching sessions were held for 4 months, in July through October 2019, scheduled at four different times during each month to accommodate participants’ schedules. The one-hour coaching sessions were held using video conferencing for ease of participation from any location. Group as well as individual participation was facilitated, largely to accommodate the schedules of practitioners. Each session focused on a practice profile core element (i.e., Engage, Explore, Plan, Access and Advocate), with an agenda and additional resources provided relevant to the topic under discussion. Participants volunteered practice experiences and insights, and shared success stories as well as challenges, along with practice tips and resources.

#### Debriefing Session

A final, in-person debriefing session was held in the sixth month of the PWLC, in November 2019. Implementation challenges and successes were discussed. Specific attention was paid to organizational issues, and agency policies and procedures, some of which had been modified by PWLC participants in their home agencies. Participants were encouraged to plan next steps in their agency teams. Final measures were completed at the end of the session, including the overall satisfaction survey. Participants shared “most significant change stories” as well as recommendations for future initiatives in writing, in response to survey items.

### Measures

Measures are described below as they reflect demographic and background characteristics (i.e., descriptive information about participants), feasibility (i.e., engagement, satisfaction, use, helpfulness and intention to use in the future), and preliminary impact (i.e., participants’ perceptions of practice changes in work with parents).

#### Demographic and Background Characteristics

Participants completed a brief survey of demographic and background characteristics in their first contact with the PWLC team, at the orientation session or the first training session, if they were unable to attend the orientation. Items included age, gender, race/ethnicity, education, parenting status, and background characteristics of professional discipline, including years working in behavioral health/human services, current agency affiliation, years with this organization, and current job title or role.

#### Feasibility

##### Engagement

Session attendance was the proxy measure for engagement in the PWLC. Session attendance was tracked for each participant over the 6-month period for a total of nine sessions: an orientation, three in-person training sessions, 4 monthly coaching sessions, and a debriefing session. In addition, participation in (i.e., visits) and content posts (i.e., types of content) were tracked in the ParentingWell virtual hub, established in Basecamp to support project management and team communication.

##### Satisfaction

Participants rated the following statements in the debriefing session survey: “I am satisfied with the format of the PWLC;” “I found the PWLC to be applicable to my role;” “I am satisfied with the trainer(s) who led the PWLC;” “I am satisfied with my overall experience with the PWLC;” “The balance between presentations, discussion and activities fits my style of learning;” and “I would recommend the PWLC to other behavioral health practitioners.” For each item, ratings ranged from: “1 = strongly disagree,” to “7 = strongly agree,” with a neutral rating option of “4 = neither agree nor disagree.”

##### Use, Helpfulness and Intention to Use in the Future

Actual use of ParentingWell Practice Profile tools and resources was assessed using four items in the debriefing session survey reflecting the number of persons served who are parents; whether the practitioners had an interaction in the past month with a person served who is a parent; if yes, whether they talked about the topic of parenting; if yes, whether they used the skills or resources learning in the PWLC; and if yes, whether these skills or resources were helpful. Open-ended items requested examples of skills or resources used; if helpful, how they were helpful; and if not helpful, how they should be changed. Participants responded to a final overall rating regarding how likely they would be to use ParentingWell skills and resources on the job, with response options ranging from “1 = not at all likely” to “7 = extremely likely”, with a neutral option, “4 = neither likely or not likely.”

#### Preliminary Impact

Participants were asked, in the debriefing survey, to provide a written example of significant change specifically resulting from their participation in the PWLC, using a Most Significant Change story technique (Dart & Davies, 2003). Participants provided a story that epitomized the most significant change in their experience, and responded to a subsequent item requesting them to indicate why the change was significant to them or to the person served (i.e., the parent or parent-to-be).

### Analysis

Survey data were entered, compiled and analyzed descriptively using Qualtrics survey software (Qualtrics, 2019). Responses to open-ended survey items were analyzed qualitatively to identify themes. Most Significant Change Story responses were transcribed and analyzed using a framework approach (Edwards et al., 2012; Ritchie & Spenser, 1994) and Dedoose software (Leiber & Weisner, 2011), with ParentingWell Practice Profile core elements and Theory of Planned Behavior constructs providing the initial codes. Analyses were conducted by experienced, doctoral level research team members, who reviewed, discussed and agreed upon code assignments and findings.

### Human Subjects Review

The study was reviewed and approved by the university Institutional Review Board. Signed informed consent was obtained from all participants. Survey data were collected and coded with confidential study ID numbers to facilitate the comparison of pre- and post-data; no participants’ names were ever associated with any data. Satisfaction data were completely anonymous, that is, not linked to study ID or participants’ names in any way. A stipend was offered to each mental health agency sending practitioners to the learning collaborative sessions, to acknowledge staff time and productivity dedicated to participation in the PWLC. The Department of Mental Health, the state mental health agency, was unable to accept a research stipend, due to state policy guidelines. Continuing education credits were offered to eligible participants (i.e., social workers and mental health counselors). There are no known conflicts of interest to report. All authors certify responsibility for the manuscript.

## Results

Study findings are reported as they relate to participants’ characteristics and to the research questions. Results, other than for the variable of engagement, are self-report data.

### Participants

Twenty-nine of a total of 30 participants completed the initial demographic and background survey. As indicated in Table 1, the majority of the participants were female (75.86%) and White (93.10%). The majority (72.41%) indicated that they were a parent or expected to become a parent in the future. More than half of the participants (58.62%) held a Master’s degree or higher; 82.75% held at least an Associate’s Degree (see Table 2). The majority of these were in human services (e.g., social work, psychology, mental health counseling). Two-thirds of participants were employed by community-based human service agencies, with a range of years of service in these agencies. Job titles and roles varied, with peer specialists representing 31% of participants.

**Table 1:** ParentingWell learning collaborative participants’ demographic characteristics (n = 29)

	% (n)
Age (n = 29)	
22 to 34	27.59% (8)
35 to 44	31.03% (9)
45 to 54	31.03% (9)
55 to 64	6.90% (2)
Gender (n = 29)	
Male	24.14% (7)
Female	75.86% (22)
Race (n = 29)	
White	93.10% (27)
Black or African American	3.45% (1)
Chose not to answer	3.45% (1)
Ethnicity (n = 29)	
Hispanic or Latino	0% (0)
Educational attainment (n = 29)	
High school or GED	3.45% (1)
Partial college credit	13.79% (4)
Bachelor’s degree	6.90% (2)
Associate’s degree	17.24% (5)
Master’s degree	58.62% (17)
Are you a parent or expecting to become a parent? (n = 29)	
Yes	72.41% (21)
No	27.59% (8)

**Table 2:** ParentingWell learning collaborative participants’ background characteristics (n = 29)

	% (n)
In what discipline is your most advanced degree? (n = 23)	
Social work	47.83% (11)
Psychology or mental health counseling	17.39% (4)
Other human services discipline	21.74% (5)
Other discipline (not in human services)	13.04% (3)
How many years have you worked in behavioral health/human services? (n = 29)	
1 to 3 years	17.24% (5)
4 to 7 years	27.59% (8)
8 to 11 years	27.59% (8)
12 or more years	27.59% (8)
Which agency or organization are you currently affiliated with? (n = 29)	
Massachusetts Department of Mental Health	34.48% (10)
Another agency or organization	65.52% (19)
How many years have you been with this agency or organization? (n = 29)	
1 to 3 years	41.38% (12)
4 to 7 years	27.59% (8)
8 to 11 years	10.34% (3)
12 or more years	20.69% (6)
What is your current job title or role at your agency or organization? (n = 29)	
Licensed clinician	20.69% (6)
Director or assistant director	24.14% (7)
Supervisor or team leader	13.79% (4)
Peer Services/family partner	31.03% (9)
Case manager	3.45% (1)
Nurse	3.45% (1)
Housing coordinator	3.45% (1)

### Feasibility

#### Engagement

Overall, attendance at various sessions (including orientation, training, coaching, and debriefing) supports the feasibility of the PWLC, as participants demonstrated their capacity and willingness to attend consistently. Of the 30 participants recruited to the study, 2 were agency executives who attended the orientation session but did not participate in the training or coaching sessions. Of the remaining 28, 6 were site directors or program supervisors who attended all 3 in-person training sessions and generally did not participate in coaching sessions; 4 participated in the debriefing session. Three of the remaining 22 participants moved to new positions during the initiative and, consequently, did not complete the study. Of the remaining 19 study participants, all direct service practitioners, the majority attended 8 or 9 sessions of the total 9 possible sessions (84%; 16/19). Three practitioners participated less frequently due to a change in work schedule, attendance at an agency summer picnic, or not consistently serving a parent client throughout the initiative. For the 19 direct service practitioners and 6 directors/supervisors, total attendance at all 3 in-person training sessions was 93% (70/75 possible person-sessions). Of the 5 training sessions missed by individuals (in total), the reasons provided related to client emergencies or personal medical emergencies. Of the 19 direct service practitioners, 100% attended the first coaching session, 1 participant missed the second session, 9 missed the third coaching session, and 4 missed the fourth coaching session. The majority of available participants (i.e., those who had not taken new jobs) attended the debrief session (88%; 21/24). Attendance was consistent across agency site, regardless of whether the site was receiving a stipend or not.

Activities in the PWLC Basecamp virtual hub reflected frequent, interactive engagement of trainers and participants. All participants accessed the virtual hub at some point during the PWLC, to check on meeting logistics and to indicate scheduling preferences for coaching sessions. Approximately 50% of the participants engaged in the interactive “Campfire”. A total of 111 posts were made by trainers and participants, with the majority (59% of total posts) made by participants. The greatest number of posts pertained to seeking or sharing information about resources for parents and families (39%) (e.g., community events and activities, listings of local parent support groups). Posts included descriptions of self-care projects (18%); suggestions for use and adaptation of ParentingWell tools and resources (12%); motivational quotes about recovery (7%); and parenting issues (6%). Questions about the PWLC and logistical issues were posted by both trainers and participants (18% of posts). Notes from coaching sessions were posted by the trainers, with participants responding to the experiences and suggestions described in sessions they had not attended.

#### Satisfaction

PWLC participants were highly satisfied with the collaborative. The majority of participants either agreed or strongly agreed with each of the six items (76% to 95%); means of items ratings ranged from 6.05 to 6.76. Eighty five percent of participants agreed or strongly agreed that, overall, they were satisfied with their experience. The majority of participants (76%) agreed or strongly agreed that they would recommend the PWLC to other behavioral health practitioners. The item receiving the greatest percentage of participants either agreeing or strongly agreeing was the item asking about satisfaction with the trainers (see Table 3).

**Table 3:** Participant satisfaction with ParentingWell learning collaborative (n = 21)

	Disagree/strongly disagree	Slightly disagree	Neither agree nor disagree	Slightly agree	Agree/strongly agree	Mean (S.D.)
Statement						
I am satisfied with the format of the PWLC	0% (0)	5% (1)	5% (1)	5% (1)	85% (18)	6.10 (1.02)
I found the PWLC to be applicable to my role	0% (0)	0% (0)	5% (1)	14% (3)	81% (17)	6.05 (0.79)
I am satisfied with the trainer(s) who led the PWLC	0% (0)	0% (0)	5% (1)	0% (0)	95% (20)	6.76 (0.68)
I am satisfied with my overall experience with the PWLC	5% (1)	0% (0)	5% (1)	5% (1)	85% (18)	6.05 (1.17)
The balance between presentations, discussion and activities fits my style of learning	0% (0)	5% (1)	0% (0)	14% (3)	81% (17)	6.14 (0.99)
I would recommend the PWLC to other behavioral health practitioners	0% (0)	10% (2)	0% (0)	14% (3)	76% (16)	6.10 (1.23)

#### Use, Helpfulness, Intention to Use in the Future

PWLC participants provided evidence of working with parents during the PWLC, deploying skills and resources learned in the PWLC, and finding them helpful. The largest percent of participants (34%) directly served as many as 4 parents, according to reports obtained in the debriefing session. Similar numbers of practitioners served between 5 and 9, and 10 to 14 parents (14% in each category). One-quarter of the participants served more than 15 parents, and 14% did not work directly with parents, but supervised practitioners who did. Of those who interacted with a parent in the last month, 90% talked about the topic of parenting.

The vast majority of these practitioners (95%) reported using skills or resources learned about in the PWLC, and reported that they were helpful. Examples of PW-informed activities included starting and having conversations about parenting; engaging, exploring and planning for positive change; using specific tools (e.g., the daily log); providing information about resources to parents; and providing information about ParentingWell to new staff and interns. Most participants (85%) indicated on the seven-point scale that they were “likely” or “extremely likely” to use ParentingWell skills and resources on the job (mean = 6.20; SD = 0.68).

### Preliminary Impact

Participation in the PWLC was reported to have impact on practice, parents being served, practitioners themselves, and the organizations in which they provide services. The Most Significant Change story technique provided qualitative data on practice change related to the ParentingWell core elements (i.e., the content of the practice approach and training) and reflected constructs from the Theory of Planned Behavior (i.e., the training targets).

#### ParentingWell Core Elements

Practitioners described ways in which their work with persons served was enhanced by the consideration and infusion of parent and family issues. They were more inclined to raise and address parent and family themes in conversation with persons served, with observed benefit to both parents and practitioners (Engage). Participants offered examples of the ways in which engagement with persons served was promoted when issues of parenting are raised: “…the program has made me more aware of people we support who may be parents and thinking to ask specific questions and include their role as a parent in our treatment plans and work.”

Exploring together with parents was facilitated by the use of ParentingWell tools (Explore) and, in particular, identifying parenting strengths as well as opportunities for making changes. One participant described, “Using the daily chart with parents gave them a visual of their day. Many parents were not aware of how much time they spent on their children, which left them no time to take care of themselves. As a parent, I understand how quickly this happens and how it becomes the normal way of life.” Pursuing these themes, as part of getting to know the person served, allowed another participant to shift a parent’s perspective: “Doing this work with her has really helped her to see her strengths and has helped her to believe that people who have mental health concerns can also be/are parents. It helped her to get unstuck…”.

ParentingWell tools facilitated conversations about goals and planning to achieve them (Plan). “Using the collaborative mapping tool opened discussion to find out what goals the couple would like to accomplish before trying to start a family. [We] continued the conversations to discuss steps needed to reach goals and what obstacles or barriers there are.” Practitioners were able to talk with parents about the importance of support-seeking and self-care, and to identify strategies for both (Access and Advocate). Participants worked together with parents to access and build social supports. “Working with her [the mother] we supported [her] through stress and helped build a relationship with another parent in services. Making friends she can relate to and have playdates was important.”

Notably, participants indicated that the impact of ParentingWell may span practice, organizational, and policy levels. At the practice level, one participant noted that the training made her “more aware of family dynamics and treating the family as a whole.” And at the organizational level, participants mentioned their intention to ensure that parenting conversations are a part of intake and supervision processes, “…hearing a newer staff relate concepts in supervision as an integrated part of their work. Staff was demonstrating they had absorbed the content and importance and had integrated into their work.” At the policy level, participants noted changes in their agency policies, including new rules allowing children to accompany parents in agency vehicles.

#### Theory of Planned Behavior Constructs

The PWLC was framed by the Theory of Planned Behavior (TPB). MSC stories were analyzed to investigate change in TPB constructs, the targets of training (see Table 4).

**Table 4:** Theory of Planned behavior constructs

Theory of planned behavior constructs	Representative quote
Attitudes Beliefs about outcomes associated with performing a particular behavior (Casper, 2007)	“It was significant to empower the individual to serve as the role of parent and validate the inherent challenges that are not related to mental illness.” “Providing support and acknowledging his commitment [to parenting] has been key to his growth.” “If a consumer takes care of themselves and their own mental health/recovery they are in a better position to be a better parent.” “Normalizing parenting frustrations and other feelings is beneficial to our relationship with clients.” “What [PWLC] has done is made me more aware of family dynamics and treating the family as a whole.”
Subjective norms Perceptions of how others would judge a person for performing the behavior (Casper, 2007); perceived social pressure to perform or not to perform the behavior (Jokonya, 2017)	“In my role as a Team Leader, I have supported clinical staff to increase the frequency, as well as the depth of discussion with the folks they serve about their parenting, and also the loss of the possibility of parenting, where appropriate. This has facilitated the opening of sensitive and rewarding discussion, which people served and staff have identified as deeply helpful.” “The attendees to this collaborative from my agency have designed and continue to revisit the PW implementation at team meetings….” “…have encouraged staff doing intakes as well as direct care staff to discuss children [and] parenting as this is not on our current comprehensive assessments. It encompasses a part of the client’s life that has not previously been discussed. Being a parent or not has helped define a part of who they are – including dreams…feelings of sadness, regret, anger, etc.”
Perceived behavioral control Perception of ease or difficulty in performing the behavior; reflects past experience as well as anticipated impediments and obstacles (Jokonya, 2017)	“I think this training has opened up more areas of questioning/work. Before I might have been reluctant to delve into the topic of parenting.” “[participant benefitted from]…approaches on how to start the conversation about parenting. It was comfortable to have the conversation.” “The ParentingWell training provided me with the awareness that my own parenting journey in my recovery is a crucial part of the process. It also made me aware that my own perceived lack of success in this area was prohibiting me from growing and helping others. By talking about this more openly it has improved my own life and enriched my role with those I support.”
Intention to change practice behavior Plan to implement ParentingWell® skills, tools and resources	“…signifies openness and enthusiasm for the shift in our work from viewing people as independent adults to folks who come from a whole family system with history; willingness on part of staff to explore.” “But I am including it [ParentingWell] more in supervisions to increase others awareness of including parenting as part of the assessment and goals….” “[The PWLC]…brought my attention to an area of future growth.”
Practice behavior Use of ParentingWell® skills, tools and resources. Changes in actual policies and practice in the agency setting	“Staff supported mom to develop boundaries and provided education in order to support her. This was significant because it gave focus and direction to a difficult situation…staff was able to problem solve and partner with her in a way that wasn't explored before.” “…hearing a newer staff relate concepts in supervision as an integrated part of their work. Staff was demonstrating they had absorbed the content and importance and had integrated into their work.” “I have used the daily schedule from the tools to assist in bringing order to his day and eliminate negative self-talk.” “Everyone has gotten on the same page about children and families also being allowed to be transported in our vehicles with the use of proper child safety car seats and booster seats. Team leaders were not aware of this change and there were differing ideas on agency policy…Change takes time, but when things change, it’s wonderful!.” “Through supervision, the case manager and I sorted out the normative challenges of parenting, the feeling the case manager had to want to protect and provide surveillance, and ultimately began the conversation that the individual supported could move on from case management. It was significant to empower the individual [client] to serve as the role of parent and validate the inherent challenges that are not related to mental illness.”

PWLC participants provided examples of their attitudes regarding parenting and family life as important aspects of a person’s life, and indicated that supporting individuals in their role as parent was useful in building a therapeutic relationship and in contributing to positive outcomes (i.e., *attitudes*). As one participant suggested, “Normalizing parenting frustrations and other feelings is beneficial to our relationship with clients.” Another explained that “providing support and acknowledging his commitment [to parenting]” was key to a client’s growth. As taking on the topic of parenting became a part of participants’ practice, they influenced the norms and expectations in their agencies (i.e., *subjective norms*). One participant reported, “…I have supported clinical staff to increase the frequency as well as the depth of discussion with the folks they serve about their parenting…”.

Participants indicated that the training resulted in them feeling more comfortable talking about parenting and family life with clients, increasing their perception of their ease in having these conversations (i.e., their *perceived behavioral control*). As a participant concluded, “I think this training has opened up more areas of questioning/work. Before I might have been reluctant to delve into the topic of parenting.” Another participant reflected on the impact of the PWLC on their understanding and integration of their past experiences into current practice. “It also made me aware that my own perceived lack of success in this area was prohibiting me from growing and helping others.” PWLC participation contributed to practitioners’ *intention to change practice behavior* as well as to make actual changes. A participant, in her role as supervisor, indicated, “…I am including it [ParentingWell] more in supervisions to increase others’ awareness of including parenting as part of the assessment and goals.” Participants reported not only actual changes in their practice behavior, but preliminary evidence of the impact of ParentingWell practice on parents themselves. “I have used the daily schedule from the tools to assist in bringing order to his day and eliminate negative self-talk.”

## Discussion

This study sought to assess the feasibility and preliminary impact of the ParentingWell Learning Collaborative as reported by practitioners regarding their own experience and the experiences of parents. The results provide preliminary evidence that the learning collaborative model is a feasible way to train and coach diverse practitioners in a new practice approach. Participants were satisfied with the training, provided evidence of embracing the content of ParentingWell core elements, and reported using skills or resources learned about in the PWLC. For example, participants engaged in conversations about parenting with their clients, provided parenting resources to them, and shared information about ParentingWell with their colleagues. The majority of participants indicated they were likely or extremely likely to use ParentingWell skills and resources in the future, suggesting that framing the PWLC in the context of the Theory of Planned Behavior contributed to participants’ intention to change practice behavior in a more family focused way. Study findings support the conclusion that further efficacy and effectiveness testing is warranted.

Alongside this support for the feasibility and preliminary impact of ParentingWell and the learning collaborative model, important considerations regarding feasibility remain. Trainer capacity is key; participants were more likely to be satisfied with the trainers than with any other component of the collaborative (although satisfaction was at least 76% with each component). Given the extremely high level of satisfaction with the trainers in the current study (95%), it is imperative to understand how to ensure that each trainer is well equipped to implement the initiative. While data from the current study suggest significant engagement with and impact from ParentingWell, it is not clear whether similar results would be obtained if satisfaction with the trainers was not so high.

A second consideration related to feasibility is the consistency of attendance. While the majority of participants attended each training session, attendance was lowest at the third coaching session, perhaps reflecting the increase of clinical workload typically associated with the month of September when, in the US, people have returned from vacation. This underscores the value of the virtual hub, which might be even more relevant as the world has grown accustomed to virtual meeting, learning and collaborating in the context of the COVID-19 pandemic. Future research should explore the potential to engage participants meaningfully in virtual, remote ways. For example, it would be helpful to identify the optimal balance between remote and in-person programming, and to ascertain whether a fully online training approach could be effectively implemented.

In addition to providing support for the feasibility of the learning collaborative model, this study suggests that the learning collaborative model can have multifaceted impact. Critically, the changes that participants mentioned differ with regard to the resources that will be necessary to implement the change. Some changes are low-cost in terms of time and money. For example, one participant noted that her agency would now allow children to accompany parents in transportation vehicles provided by the agency. This policy change could be made immediately and did not require any further investment. Other changes are broader and more long-term as they involve organizational culture change. Examples of this type of change include using ParentingWell tools and resources in training of interns and junior staff, and ensuring that parenting conversations are part of intake and supervision processes. The finding that staff have already implemented relatively simple changes is promising, yet continued support and commitment from organizational leadership may well be necessary to achieve organizational culture change. Additional research is needed to assess the extent to which organizational leadership pursued these longer-term initiatives. Future research can also identify other avenues of impact. For example, it is possible that the ParentingWell Learning Collaborative, as it helps professionals to feel more effective, will also contribute to their job satisfaction, perhaps even resulting in longer tenure in their roles. While it is not possible to draw these conclusions from this study’s data, these are important considerations for future research. Also, while practitioners described ways in which their participation with the ParentingWell Learning Collaborative impacted parents, additional research is needed to identify and measure specific parent outcomes.

It is also important to acknowledge that the learning collaborative approach was costly, as it required a significant time commitment from trainers and participating agencies. Four of the five participating agencies received a stipend for practitioners to participate. Realistically, to overcome barriers to training, both practitioners as well as organizations must be incentivized (e.g., continuing education credits for practitioners and agency stipends to cover lost productivity). It is possible that the development of online teaching strategies can mitigate some of these costs, while also overcoming geographic barriers to in-person training (e.g., transportation and costs associated with a meeting venue). Also, while participants were incentivized through continuing education credits and agencies were provided stipends, there was evidence of intrinsic motivation. There was no compensation for participation on the virtual Basecamp, yet participants contributed to these discussion boards consistently and enthusiastically. Also, even though we were precluded from providing a stipend to the Department of Mental Health, their staff members were positively engaged throughout the ParentingWell Learning Collaborative. This likely reflects high levels of intrinsic motivation to engage with the ParentingWell Learning Collaborative.

### Limitations

This study provided solid preliminary evidence for the feasibility and the impact of the ParentingWell Learning Collaborative. However, the study has limitations that future research should address. First, as a pilot study, this study had a small sample of practitioners. The small sample size precluded our ability to systematically investigate differences between professional groups, and these potential differences comprise an important consideration for future study (Maybery et al., 2014; Skogoy et al., 2019). Also, the measures used in the current study are administered via self-report and not standardized; the development of a standardized outcome measure for training impact and ParentingWell practice is a critical next step. Future research should incorporate longer-term engagement, sustainability and follow-up. Many of the participants mentioned intention to implement broad initiatives within their organizations that would require organizational culture change to infuse parenting considerations throughout the organization’s practices and policies. While the framing of intentions by themselves indicate that the ParentingWell Learning Collaborative impacted how participants thought about these issues, it is not yet known whether intentions will translate to actual change.

A second critical limitation is the homogeneity of the sample in the current study. Approximately 93% of the participants were White and approximately 76% of the participants were female. This is likely reflective of the population of behavioral health practitioners in public sector, community-based agencies in Massachusetts but is still problematic, given the widely accepted notions that parenting values and behaviors are gender-influenced and culturally bound. Addressing parenting and family life within the mental health service system requires a workforce that is diverse and culturally competent. Despite the lack of diversity along gender, racial and ethnic categories, the sample was diverse with regard to training, job title, and job responsibilities, suggesting that a well-articulated practice approach to parenting and family life is accessible and useful to a range of practitioners.

## Conclusions

The experiences and needs of parents with mental illness are well-studied. However, their needs are infrequently addressed. Increasing the capacity of adult mental health service providers to work in family-focused, parent-informed ways has the potential to significantly improve outcomes for both adults and their children. This requires the development of practice approaches, intervention and program models that are theoretically- and empirically sound, and accessible and useful to the range of practitioners in diverse service settings. The rigorous testing of these interventions and the assessment of outcomes for parents and families depend on the implementation of these models with fidelity to core program elements and the adaptation with care in additional contexts. The training and coaching of practitioners, an often-neglected step in the implementation and adaptation of interventions, are key to rigorous research, the demonstration of impact, and the sustainability of effective models. The ParentingWell Practice Profile and Learning Collaborative model are steps in this direction for parents with mental illness and their families, and the practitioners who work with them.

## Data Availability

The ParentingWell Practice Profile, tools and resources are available on the website of the National Research Center for Parents with Disabilities at Brandeis University (https://heller.brandeis.edu/parents-with-disabilities/training/parenting-well/index.html) and the Massachusetts Department of Mental Health Children’s Behavioral Health Knowledge Center website (https://www.cbhknowledge.center/parenting-well). Study data are available from the lead author on request.
